# Research progress on the neutrophil components and their interactions with immune cells in the development of psoriasis

**DOI:** 10.1111/srt.13404

**Published:** 2023-07-13

**Authors:** Zhenhui Wang, Dongmei Shi

**Affiliations:** ^1^ Shandong University of Traditional Chinese Medicine Jinan Shandong China; ^2^ Chief Physician, Doctoral Supervisor, Department of Dermatology & Laboratory of Medical Mycology Jining No. 1 People's Hospital Jining Shandong Province China

**Keywords:** granule proteins, neutrophil, neutrophil extracellular traps, psoriasis

## Abstract

**Background:**

Psoriasis is an immune‐mediated chronic inflammatory disease, and currently it is widely believed that the IL‐23/IL‐17 axis and Th17 cells play a critical and central role. However, increasing evidence suggests that neutrophils may interact with a variety of immune cells to play an indispensable role in psoriasis.

**Materials and methods:**

We searched the recent literature on psoriasis and neutrophils through databases such as PubMed and CNKI, and summarized the findings to draw conclusions.

**Results:**

Neutrophils can promote the development of psoriasis by secreting IL‐23, IL‐17, and cytokines with TH17 cell chemotaxis. Activated keratinocytes (KCs) can attract and activate neutrophils, induce the formation of neutrophil extracellular traps (NETs). KCs can also expose self‐antigens which lead to strong autoimmune reactions. The granule proteins secreted by activated neutrophils can activate IL‐36, which converts vulgaris psoriasis to generalized pustular psoriasis (GPP).

**Conclusion:**

The function of neutrophils components and the interaction between neutrophils and immune cells play an essential role in the pathogenesis of psoriasis. The aim is to provide a theoretical basis for the exploration of targeted clinical treatments and fundamental research on the pathogenesis of psoriasis.

## 1 INTRODUCTION

The incidence of psoriasis accounts for about 2% of the world's population,^1^, ^2^ affecting about 120 million people.^3^ In China, the incidence is 0.47%, and it is estimated that there are about 6.5 million patients in China, with a trend of increase.^4^ Psoriasis is an immune‐cell‐mediated chronic systemic inflammatory disease that mainly affects the skin.^1^ Psoriasis can be divided into plaque‐type psoriasis, pustular psoriasis, erythrodermic psoriasis, arthritis psoriasis, and so on.^1^ Psoriasis can be accompanied by various other diseases, such as atherosclerosis, chronic obstructive pulmonary disease, inflammatory bowel disease, type 2 diabetes, and kidney disease.^1^, ^5^, ^6^ Previous studies have suggested that Th17 cells and the IL‐23/IL‐17 axis play a critical role in the pathogenesis of psoriasis.^7^ Currently, biologic agents that antagonize TH17 cell‐related cytokines, such as Tumor necrosis factor (TNF) antagonists,^8^ IL‐17 antagonists,IL‐23 receptor antagonists, IL‐23 antagonists, and IL‐36 antagonists, have been developed.^9^ They can quickly clear skin lesions and bring a qualitative leap in the treatment of psoriasis,^10^ but they can only achieve clinical remission. Some patients still experience relapse or worsening during or after treatment, which suggests that other factors may be involved in the mechanism of psoriasis development.

**FIGURE 1:**
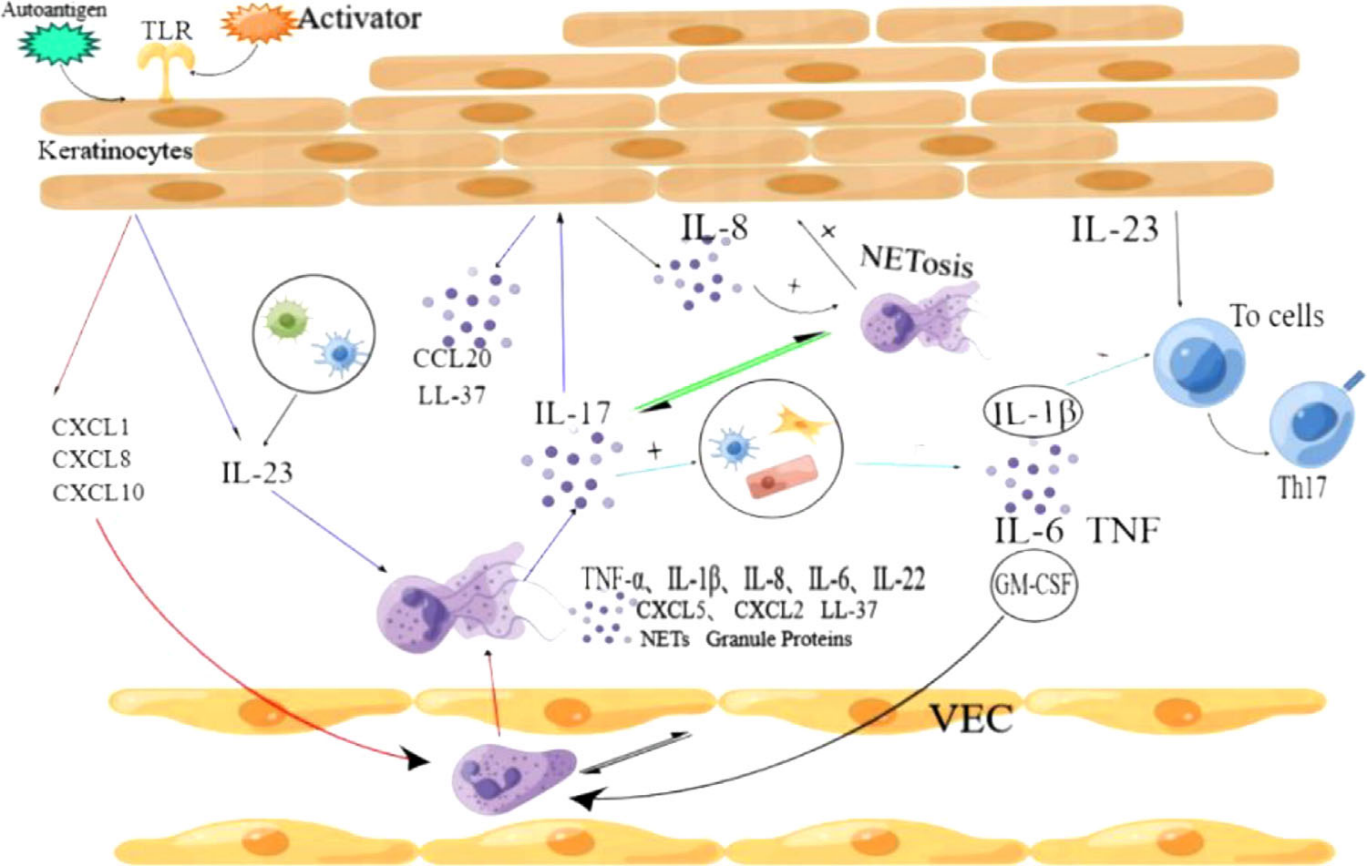
(1) Toll‐like receptor 3 (TLR3) on the membrane of keratinocytes (KCs) cells can be activated by activator (double‐stranded RNA), leading to the release of IL‐23. KCs can also be stimulated by other activators to release neutrophil‐derived chemokines, thereby attracting and activating neutrophils. (2) Activated neutrophils can produce TNF‐α, IL‐1β, IL‐8, IL‐6, IL‐22, CXCL5, CXCL2, LL‐37, NETs, granular protein, and so on. (3) There is a mutual activation between neutrophils and vascular endothelial cells (VECs). (4) IL‐17 can induce dendritic cells, epithelial cells, and fibroblasts to produce IL‐1β, IL‐6, TNF, and GM‐colony‐stimulating factor (CSF). (5) IL‐17 and IL‐8 promote The formation of extracellular traps by neutrophils (NETosis), and NETosis can release more IL‐17. (6) Products formed during neutrophil NETosis can also promote the proliferation and activation of KCs. (7) VECs and neutrophils have a mutual activation effect. (8) IL‐1β and IL‐23 can both induce the differentiation of initial naive T cell (T0 cell) into TH17 cells.

Neutrophils are the most abundant cells of innate immunity and the most prevalent white blood cells in the blood of healthy individuals. They play an indispensable role in anti‐infective and inflammatory processes through mechanisms such as antigen presentation, reactive oxygen species (ROS) generation, release of granular proteins, formation of neutrophil extracellular traps (NETs), and production and release of cytokines.^11^, ^12^, ^13^ Changes in neutrophils within the skin lesions in patients with psoriasis suggest that neutrophils play an important role in the development of psoriasis. KCs, the skin's epithelial cells, release chemokines such as CXCL1, CXCL8, CXCL10, CCL2, CCL3, CCL5, and CCL20. Those chemokines attract neutrophils to psoriatic lesions.^14^ Neutrophil‐keratinocyte interactions can result in the production of IL‐17, which initiates or exacerbates the skin lesions^15^ (Figure 1). Recent studies have found that neutrophils are responsible for the most IL‐17 production in psoriatic lesions.^16^ Suggesting that neutrophils play a key role in the development of psoriasis. The roles of IL‐17/IL‐23 axis have been widely reviewed otherwise for psoriasis,^7^ we will focus on the new insights on the function of neutrophils and their potential mechanisms of action in interacting with other immune effectors during the pathogenesis of psoriasis in this review. We hope it will give a clue for exploring new treatment strategies for psoriasis in future.

### 1.1 Neutrophil cytokines and psoriasis

Neutrophils, as a type of innate immune cell, are the first to reach the site of inflammation in the early stages of inflammation. Neutrophil lifespan is short, and apoptosis begins after entering the blood for 24 h, but during the period of psoriasis inflammation, the lifespan of neutrophils can be significantly extended.^13^ For example, during inflammation, KCs can prolong neutrophil lifespan.^17^ The chemokines released by skin KCs can attract neutrophils to migrate constantly to psoriasis lesions and prolong neutrophil lifespan during inflammation.^17^ And then, neutrophils produce and secrete IL‐17^16^ and other cytokines, which can participate in the occurrence and development of psoriasis.

#### 1.1.1 IL‐23

**FIGURE 2:**
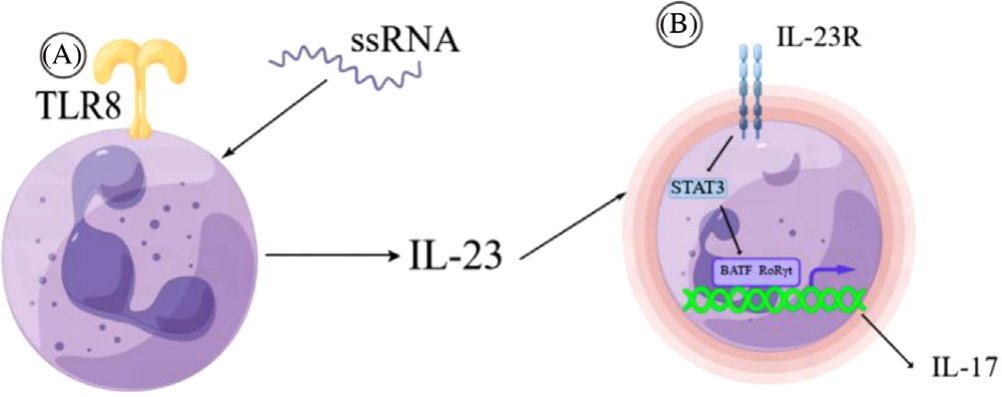
(A) Single‐stranded RNA (ssRNA) can activate the toll‐like receptor 8 (TLR8) receptor on the surface of neutrophils, promoting their production of IL‐23. (B) IL‐23 induces neutrophil production of IL‐17 through the STAT3‐dependent RORγt and BATF pathways. The ability of neutrophils to produce both IL‐23 and IL‐17 results in a pro‐inflammatory positive feedback loop.

IL‐23, composed of subunits p40 and p19. Macrophages, dendritic cells, and KCs in psoriatic skin tissue can secrete IL‐23^7^, ^18^, ^19^; and neutrophils in the blood of healthy individuals also express IL‐23.^20^ Membrane toll‐like receptor 8 (TLR8) on the surface of Neutrophil can be activated by single‐stranded RNA (ssRNA), resulting in promoting IL‐23 production by neutrophil^21^ (Figure 2). This type of RNA may originate from viruses or NETs, indicating that viral infection may exacerbate psoriatic skin lesions.

After binding to IL‐23R, IL‐23 can exert various biological effects. IL‐23R is expressed on the surfaces of lymphocytes and myeloid cells (dendritic cell, macrophage, and monocyte).^22^ The binding of IL‐23 to IL‐23R promotes immune cell activation, induces the formation of mast cell extracellular traps (MCETs), induces the production and release of IL‐17 and chemokines, and promotes the differentiation of naive T cell into Th17 and Th1 cells^16^, ^23^ (Figure 1). Recently, it was found that neutrophils also express IL‐23R,^24^ and the release of IL‐23 by neutrophils can bind to its surface receptor IL‐23R, which lead to self‐activation of neutrophils and further exacerbate inflammation in psoriasis (Figure 2). Alternatively, IL‐23‐activated neutrophils may be capable of generating NETs, similar to mast cells (Figure 1). This remains to be further explored.

#### 1.1.2 IL‐17 produced by neutrophils

It is currently believed that IL‐17 plays an important role in psoriasis, and IL‐17 antagonists such as secukinumab have been widely used in clinical practice.^25^ The IL‐17 cytokine family consists of IL‐17A, IL‐17B, IL‐17C, IL‐17D, IL‐17E, and IL‐17F, among which IL‐17A and IL‐17F have the strongest pro‐inflammatory effects.^26^ IL‐17A can be produced by T cells, innate lymphoid cells, mast cells, and neutrophils. In the pathogenesis of psoriasis, the Th17 cell is considered to be the main producer of IL‐17.^15^ However, increasing evidence shows that neutrophils are the most abundant cell type producing IL‐17 in psoriatic lesions.^27^, ^28^ Studies have demonstrated that IL‐23 can specifically promote the transcription‐level production of IL‐17A, IL‐17F, and IL‐22 by neutrophils. IL‐23 induces to produce IL‐17 through the STAT3‐dependent RoRγt and BATF pathways by neutrophil.^24^ (Figure 2). The IL‐17R, IL‐17 receptor, is expressed on monocytes, lymphocytes, fibroblasts, and KCs.^29^ IL‐22 promotes the proliferation and hyperkeratination of KCs.^30^ The function of IL‐17A in autoimmune disorders is to promote the release of cytokines, chemokines, and matrix metalloproteinases from tissue cells,^31^ and to enhance the production of antimicrobial peptides and chemokines by KCs.^32^ IL‐17A has been shown to drive the production of IL‐1β, IL‐6, TNF, and GM‐ colony‐stimulating factor (CSF) by fibroblasts,^33^ epithelial cells,^34^ and dendritic cells.^35^ Among these factors, IL‐1β and IL‐17A cooperatively promote Th17 cell proliferation^36^; IL‐17A can also induce the expression of chemokines CXCL2 and CCL20.^37^ Although neutrophil recruitment is mainly influenced by the CXCL8 chemokine family,^38^ IL‐17A can induce the release of granulocyte colony‐stimulating factor (G‐CSF) and GM‐CSF to promote the proliferation and survival of neutrophils,^39^ which further recruit neutrophiles at the inflammatory site and resulting in a self‐amplifying effect (Figure 1).

Among other diseases comorbid with psoriasis, IL‐17‐producing neutrophils have also been observed. IL‐17+ neutrophils were found in atherosclerotic plaques.^40^ These IL‐17+ neutrophils may be one of the mechanisms behind the comorbidity of psoriasis with other diseases.

### 1.2 The function of chemokines produced by neutrophils

Neutrophils can be recruited by various chemokines and migrate to the lesion site. KCs can produce neutrophil chemokines, but previous studies have shown that neutrophils themselves can also produce chemokines under the stimulation of inflammation,^47^ resulting in a self‐amplifying effect. Moreover, this study showed that not only neutrophils, but also monocytes, macrophages, dendritic cells, NK cells, and T cell subsets can be recruited by neutrophil‐produced chemokines, including Th17 cells, closely related to psoriasis. This reflects the central role of neutrophils in immune inflammation.Various conditions can induce neutrophils to produce their own chemokines. Such as G‐CSF can induce neutrophils to produce CXCL5^48^ and CXCL2^49^. Under the stimulation of microorganisms or their derivatives such as lipopolysaccharides, neutrophils can produce CCL20 and CCL19.^50^ IL‐23 can promote the release of G‐CSF and indirectly promote neutrophil production by neutrophil. However, there is currently no evidence to confirm that IL‐23 can directly promote neutrophil production of chemokines. Further exploring whether IL‐23 can directly promote neutrophil to produce chemokines, which deserves a further study to clarify.

### 1.3 The role of neutrophil granule proteins in Psoriasis

The composition of neutrophil granule proteins is relatively complex and can be divided into three major categories, including primary azurophilic granules (myeloperoxidase (MPO) etc.), secondary specific granules, and gelatinase granules (matrix metallopeptidase 9 (MMP‐9) etc.).^51^ Different particles have different functions, and those related to psoriasis include MPO, various neutrophil proteases, MMP‐9, etc. The granule proteins of these neutrophils promote the occurrence and development of psoriasis by activating cytokines released by other cells or damaging the VECs. Elastase, proteinase 3, and tissue proteinase G are all part of the neutrophil azurophilic granules. They not only activate the IL‐36 precursor into the more forms of IL‐36β and IL‐36γ, but also inactivate IL‐36Ra through their elastase, further enhancing inflammation.^52^ In addition, it promotes the over‐proliferation of KCs in psoriatic lesions by activating the EGFR pathway,^11^ as well as promoting the secretion of type I IFN by myeloid dendritic cells.^53^ The proteinase 3 and tissue proteinase G can respectively activate neutrophils to produce chemokines CXCL‐8 and CXCL‐5.^54^ This will cause self‐amplification of neutrophil chemotaxis. MMP‐9 belongs to the specific granules of neutrophils and is involved in cell apoptosis, innate immunity, and kidney development, and also has the function of limiting bacterial proliferation.^55^ The further study demonstrates that MMP‐9 can disrupt the tight junctions and cytoskeletal integrity of endothelial cells, damage the vascular barrier function, and significantly increase vascular permeability, further enhancing inflammation.^56^ Recently, it was found that neutrophils with *MPO* gene defects have decreased ability to produce NETs.^57^ One possible mechanism is that the formation of NETs requires the participation of ROS. However, the same study also showed that the reduction of MPO leads to an increase in the activity of the serine protease (containing elastic enzyme, proteinase‐3, and cathepsin G) of neutrophils, which may result in the activation of more IL‐36. And the expression of CD47 on the cell membrane of MPO‐deficient neutrophils is increased, while CD47 can inhibit the phagocytosis of monocytes on neutrophils.^58^ Therefore, MPO deficiency may be the cause of worsening IL‐36‐mediated GPP.

### 1.4 NETs

#### 1.4.1 Generation and NETsis of NETs

NETs are fibrous networks released by neutrophils that contain various components such as neutrophil granule proteins, LL‐37, RNA, and cytokines.^59^, ^60^ Initially, NETs were believed to play a role in trapping and killing extracellular pathogens.^61^ However, NETs are not only produced during states of infection but can also be produced in response to immune complexes and cytokines such as IL‐8 and IL‐23.^62^ Indicating that NETs not only have anti‐infective effects but also play a role in autoimmunity. Eosinophils, mast cells, neutrophils, macrophages, and Th17 cells can all produce extracellular traps.^63^ Compared to healthy controls, patients with psoriasis have higher levels of NETs in their skin lesions and peripheral blood.^63^ Indicating that NETs play an important role in psoriasis.

The formation of extracellular traps by neutrophils is called NETosis.^64^ There are three modes of NETosis, the first of which is suicidal. Neutrophils triggered by activators (such as carnitine, autoantibodies or uric acid crystals)^64^ can induce ROS activation. Thus ROS activating peptidyl arginase deaminase 4, which leads to chromatin depolymerization. Neutrophil elastase and MPO are transferred to the nucleus to promote chromosome decondensation and nuclear membrane rupture. Ultimately, neutrophil death, and the release of NETs into the extracellular space.^64^ The second mode, independent of ROS, is triggered by TLR and complement C3 receptors. It involves nuclear chromatin decondensation and nuclear membrane rupture, as well as the release of nuclear DNA. However, after this type of NETosis, neutrophils are still able to phagocytose pathogens, and neutrophil lifespan is not affected by DNA loss.^65^The third mode of NETosis also depends on ROS, but results in the release of mitochondrial DNA.^66^

The uncontrolled release of NETs may contribute to various diseases such as psoriasis, systemic lupus erythematosus,^67^ rheumatoid arthritis,^68^ cardiovascular disease, inflammatory bowel disease,^69^ etc. Therefore, NETs may not only be mechanisms of psoriasis but also mechanisms of comorbidities.

#### 1.4.2 Functions of NETs

NETs contain MMP‐9,^11^, neutrophil elastase, MPO, and cathepsin G,^60^ as well as proteases 3 and LL‐37.^70^ In various autoimmune diseases, such as rheumatoid arthritis^71^ and systemic lupus erythematosus,^72^ NETs also contain self‐antigens, which can be recognized by immune cells to induce autoimmunity.^73^ The antibacterial peptide (LL‐37) can also act as a self‐antigen,^65^ and is found in many cells, especially in KCs and neutrophils,^74^ LL‐37 can form complexes with nuclear DNA or RNA produced by NETs (DNA‐LL37 or RNA‐LL37).^74^ DNA‐LL37 bind to TLR8 expressed on inactive neutrophils to produce further NETs and large amounts of cytokines, resulting in further inflammation. LL‐37 can also activate myeloid dendritic cells, Th17 cells, and KCs through TLRs to produce IFN‐β which ultimately cause further exacerbation of autoimmunity and induction of psoriasis.^75^ Although the source of RNA required for formation of RNA‐LL37 has not been confirmed for neutrophils, NETs themselves can be produced through suicidal NETosis of neutrophils, and neutrophils contain large amounts of RNA. Perhaps the release of large amounts of RNA from lysed neutrophils binds to LL‐37 to form RNA‐LL37. In addition, certain RNAs do not require formation of RNA‐LL37 to promote psoriasis formation. For example, Hua et al. pointed out that lncRNA can promote psoriasis formation.^76^

Both mast cells and neutrophils release IL‐17 during the formation of extracellular traps, but neutrophil NETs contain higher levels of IL‐17 than MCETs do.^16^ This may be one reason why neutrophils rather than mast cells are dominant in psoriasis patients. NETs release IL‐17, which in turn promotes NETosis, forming a cytokine amplification loop. This indicates that NETs can promote the onset and maintenance of psoriasis through multiple pathways.

### 1.5 The interaction of neutrophils with other immune cells

In traumatized or microbially infected skin, mast cells recognize viruses, bacteria, and other pathogens through TLRs.^84^ This may lead to mast cells releasing TNF‐a, IL‐17, and CXCL2 through degranulation or MCETs.^16^ These mediators cause an increase in vascular permeability and the chemotaxis of neutrophils to the site of inflammation. IL‐23 produced by KCs^85^ can rapidly induce mast cell degranulation and extracellular trap formation, promoting the release of IL‐17 by mast cells, etc. Mast cells, which are already resident in skin tissue, may reflect a synergistic interaction with neutrophils in early psoriatic skin. And may be one mechanism of the Koebner phenomenon in psoriasis patients. Interactions also exist between IL‐17‐producing T cells and neutrophils, as IL‐17‐producing T cells can produce cytokines that promote neutrophil development, recruitment, and survival.^13^

KC in the skin has antigen‐presenting function.^86^ Streptococcus is believed to be associated with psoriasis,^87^ the antigen produced by Streptococcus can be presented by KC to T cells, thereby triggering psoriasis. Some studies have shown that KC's keratin^87^ or carbohydrates^88^ have common antigens with Streptococcus. After skin infection with Streptococcus, autoantibodies are formed. And due to genetic susceptibility, KCs are more likely to respond to damage, thus initiating psoriasis. In the initial stage of psoriasis, KCs also produce antimicrobial peptides (AMPs), such as β‐defensin^89^ and LL‐37.^90^ Among them, LL‐37 is recognized as the main autoantigen of psoriasis,^91^ which ultimately induces Th17 cells to produce IL‐17^92^ through a series of reactions. Increasing evidence shows that KCs can produce IL‐23,^19^, ^28^, ^93^ triggering a cascade reaction (Figure 1). One of the pathways leading to the release of IL‐23 by KCs is the activation of TLR3, which can be dependent on double‐stranded RNA (dsRNA).^93^ DsRNA can come from viruses or from the organism.^94^ Perhaps dsRNA is a autoantigen that can induce psoriasis. Therefore, perhaps in the early stage, psoriasis is independent of Th17. As keratinocytes produce IL‐23, mast cells originally stationed in the skin tissue will release IL‐17 first.^16^ Or in the early stages of inflammation, neutrophils arrive at the skin first, causing a series of reactions. This is because KCs simultaneously express and release both neutrophil chemokines and IL‐23, allowing neutrophils to be chemotactically activated. Studies have shown that co‐incubation of KCs and neutrophils for 4 hours can induce neutrophils to produce IL‐17 and IL‐22.^15^ We can speculate that IL‐23 activates the IL‐23R on neutrophil membranes, causing neutrophils to release IL‐17. And IL‐17A is one of the most pro‐inflammatory cytokines in the IL‐17 family, which binds to the receptor IL‐17R on KCs, leading to an increase in the production of cytokines such as TNF‐α, IL‐1, IL‐8, and IL‐6^95^ (Figures 1 and 2). Neutrophils can also induce KC proliferation and keratinization by producing IL‐22.^30^ IL‐17 can also stimulate KCs to produce human β‐defensin 2 and other AMPs (including LL‐37).^30^ Studies have shown that IL‐19 is downstream of the IL‐23/IL‐17 axis, and IL‐19 can be secreted by KCs.^42^ This self‐secreted IL‐19 by KCs leads to keratin layer proliferation. If KCs can produce more IL‐36 when activated by IL‐17,^45^ this may lead to a more severe type of psoriasis.

When activated by pathogenic factors in the internal and external environment, KCs release neutrophil‐derived chemokines, which results in the recruitment of neutrophils to the skin. When neutrophils arrive at the vessels in the skin, they interact with the VECs. The MMP‐9 produced by neutrophils destroys cell connections between VECs, causing vascular dilatation and increased permeability. Along with changes on vascular endothelial cells, neutrophils are activated,^96^ and lifespan and migratory ability of neutrophils are increased.^17^ Activated neutrophils form NETs, causing the release of additional inflammatory factors. On the other hand, neutrophils mainly adhere to atherosclerotic plaques through the formation of NETs.^97^ The IL‐17 produced by activated neutrophils acts in synergy with TNF‐α to stimulate endothelial cells to express neutrophil‐derived chemokines (CXCL1, CXCL2, and CXCL5),^98^ attracting more neutrophils to the affected area. This interaction between skin VECs and neutrophils further exacerbates the inflammation in psoriasis, and may explain why patients with psoriasis are prone to cardiovascular diseases. After migrating to the skin tissue, neutrophils promote the progression of psoriasis by producing IL‐23, IL‐17, granule proteins, NETs, and releasing other cytokines.

## 2 CONCLUSION AND PROSPECT

Neutrophils are innate immune cells that play a critical role in the early stages of psoriasis inflammation. They can interact with other immune cells such as KCs and mast cells through the production of IL‐17, formation of NETs, and release of granule proteins. Neutrophil lifespan is significantly extended, and KCs and TH17 cells can attract neutrophils, amplifying the inflammatory effect.

Previous studies have identified the role of NETs in psoriasis, atherosclerosis, uremia, inflammatory bowel disease, and chronic obstructive pulmonary disease. NETs also play an important role in the pathogenesis of comorbidities. Today several drugs that can inhibit NETs have been developed for possible blocking NETosis, including cannabidiol [99],^99^ acting on the formation and the pro‐inflammatory components within NETs. NETosis inhibitor ascomycin,^100^ and antagomiR‐155 that can inhibit PAD4 (an enzyme involved in NETosis).^101^ We expected that their applications through combination with current biologicals or alone would provide more clinical solutions for psoriasis and comorbidities.The lifespan of activated neutrophil is then significantly extended with these effectors. KCs and TH17 cells can attract neutrophils, amplifying an overall inflammatory effect in psoriasis.

## CONFLICT OF INTEREST STATEMENT

The authors have no relevant financial or nonfinancial interests to disclose.

## Data Availability

The data that support the findings of this study are available from the corresponding author upon reasonable request.
